# Influence of Faculty-Derived Concept Maps on Student Study Strategies

**DOI:** 10.1007/s40670-025-02496-4

**Published:** 2025-09-02

**Authors:** Kaitlin P. Hardy, Amanda K. Burbage, Julie A. Kerry

**Affiliations:** 1 Macon and Joan Brock Virginia Health Sciences Eastern Virginia Medical School at Old Dominion University, Norfolk, VA USA; 2 Department of Medical and Health Professions Education, Macon and Joan Brock Virginia Health Sciences EVMS School of Health Professions at Old Dominion University, Norfolk, VA USA; 3 Department of Biomedical and Translational Sciences, Macon and Joan Brock Virginia Health Sciences Eastern Virginia Medical School at Old Dominion University, Norfolk, VA USA; 4 Children’s Hospital of the King’s Daughters, Norfolk, VA, USA

**Keywords:** Concept mapping, Active learning, Cognitive integration

## Abstract

**Background:**

Concept mapping is a well-established tool for students to actively organize information into a visual and spatial framework. However, some students are resistant to adopting concept mapping as a study strategy due to the time and effort involved in generating the maps. In this study, we explored student perceptions of faculty-derived concept maps (FD-maps) developed to accompany content within a Foundational Science module in a medical school curriculum. We subsequently investigated whether student use of FD-maps helped overcome resistance to adoption of concept mapping.

**Methods:**

Student evaluation data was analyzed using a mixed methods approach to explore student perceptions of FD-maps. Based on this analysis, we deployed a survey to M1 (26% response rate) and M2 students (20% response rate) to assess the impact of FD-maps on subsequent use of concept mapping. Data from the survey was analyzed by binary logistic regression, and the Wald test was used to analyze predictor variable effects on the outcome.

**Results:**

Students perceived the FD-maps as helpful, allowing them to streamline their studying and helping them organize and make connections between the module content. Survey analysis found that overall student use of concept mapping increased after completion of the Foundational Science module. In addition, ranking the importance of the FD-maps in their studying and annotation of the FD-maps increased the adoption of concept mapping in subsequent modules.

**Conclusions:**

Students perceived FD-maps as helpful to their learning, and their utilization led to increased implementation of concept mapping by students. Thus, FD-maps may partially aid in overcoming barriers to students adopting concept maps as part of their overall study strategies.

## Introduction

The foundational sciences taught in the preclinical phase of medical education form the basis of later application of knowledge in the clinical years and beyond [1–3]. However, there is a gradual loss of core basic science knowledge over time as individuals progress in medical school and eventually into practice [4–8]. Significant efforts have been made to improve instructional and study strategies for medical students to enhance long-term retention, including active learning and integration. Active learning includes modalities such as Team-Based and Case-Based Learning and consistently produces better retention of information than passive learning approaches [9–13]. Integration can take multiple forms—as medical schools have moved to shorter periods of preclinical instruction, there has been a corresponding switch to integrated or systems-based curricula where the traditional discipline boundaries are diminished if not eliminated [14]. Vertical integration takes this concept a step further where the preclinical and clinical sciences are viewed as a continuum [15, 16]. Finally, cognitive integration incorporates the explicit linkage of relationships between foundational knowledge and clinical features to increase retention and application of knowledge [9, 17, 18]. By promoting meaningful learning, cognitive integration approaches result in knowledge retention that is stronger and longer lasting than rote memorization, as students are encouraged to address the conceptual meaning of new knowledge then link this new information with their prior knowledge base.

One method of active learning that can facilitate cognitive integration and meaningful learning is concept mapping [19–23]. Concept mapping is a visual and spatial education tool that encourages meaningful, active learning through the relation and integration of concepts. Rooted in assimilation theory as it applies to meaningful learning [24, 25], concept mapping replaces unidirectional organization with thinking that proceeds in multiple directions [26]. Concept mapping is widely used throughout medical education, and its efficacy in a broad sense can be ascribed to four main properties: promoting meaningful learning, providing learning resources, providing feedback to students, and as assessment of learning [20, 22, 23]. Concept maps have been shown to improve retention and exam performance in disciplines fundamental to medical curricula such as biochemistry [27, 28] and specific topics such as cardiovascular therapeutics and physiological homeostasis [29, 30]. They promote the visual display of pathophysiological reasoning and learning rooted in clinical vignettes [31], can be integrated into problem-based learning [32], and have been shown to enhance critical thinking [33]. However, studies have also identified barriers to students adopting concept mapping as a learning approach, notably the time that it takes to create the maps as they are normally student-generated [34]. In addition, a significant percentage of students find concept mapping to be difficult, time-consuming, and not helpful for their learning [35, 36].

In the current study, we describe the use of faculty-derived concept maps (FD-maps) as part of a Foundational Science module that included the topics of Biochemistry, Cell Biology, and Immunology. The structure of the module was designed in alignment with cognitive load theory, incorporating strategies to reduce extraneous load and manage the intrinsic load, such as chunking of information [37–40]. The FD-maps were provided for all learning content within the module as pre-developed schema, with the goal of aiding connections of the schema within the learner. The FD-maps were designed as “skeleton” maps—essentially a “cliff notes” version of the learning content that provided space for the learners to annotate the maps by adding additional information and linkage words. We initially set out to assess student perceptions of the FD-maps and gain insight into how students used the FD-maps. To address this question, we analyzed data from student evaluations across 4 years of the module using both quantitative and qualitative approaches. In addition, we sought to determine the extent that FD-maps impacted student perception and use of concept mapping using a survey tool. Our hypothesis was that the use of FD-maps would positively influence continued use of concept mapping as a learning strategy beyond the Foundational Science module and may indicate a means to overcome an important barrier to student use of concept mapping as a learning strategy.

## Materials and Methods

This study utilized a two-phased sequential observational mixed methods approach. In the first phase, we analyzed student end-of-module evaluations to evaluate perceptions of the FD-maps by students completing the Foundational Science module between 2016 and 2020. Based on these findings, we wished to determine whether FD-maps influenced student adoption of concept mapping in subsequent modules. A survey was developed to address this question that was administered to a single cohort of M1 and M2 students in 2021. IRB approval for the survey was sought and approved as exempt (IRB # 20-10-XX-0190).

### Design of the Foundational Science Module

The Foundational Science module was designed according to the principles of cognitive load theory, including overview lectures to provide contextual and meaningful learning, chunking of detailed information into short, interactive “Learning Units” and regular active learning opportunities to review and apply knowledge. All learning materials were accompanied by FD-maps as pre-developed schema with the intent that students would use the maps as a framework to learn and connect concepts. The maps, referred to in the curriculum as “study guides,” included all the lecture keywords and high-yield information but lacked linkage words and included sufficient space for students to add their own linkage words and additional annotations (see Fig. 2 for an example). All students receive an introduction to concept mapping as a learning tool prior to the start of the module. The Foundational Science module was implemented in the first year of the MD curriculum in 2016 as part of a major curriculum revision known as the CareForward Curriculum at Eastern Virginia Medical School. At that time, the module included Biochemistry, Cell and Molecular Biology and Genetics. In 2018, the module was modified to include Cell Physiology, and the Foundations of Immunology and FD-maps were included for all new module content.

### Qualitative Data Collection and Analysis

Data from annual student evaluations was collected anonymously by the Office of Assessment and Evaluation. All students in the class are required to complete the evaluations (between 150 and 155 students per year) and all evaluations in the period between 2016 and 2020 included a variation of the following question: “Describe the three best experiences in this module.” Not all students answered each question within the evaluation; therefore, the response rate to this question ranged from 97% in 2016 to 43% in 2020. The frequency of all responses, including those that mentioned the FD-maps, was noted as a percentage of total responses to the question. Subsequently, the student evaluation responses from 2016 to 2020 were subjected to a thematic analysis using an inductive approach [41]. Initially, the 2016 and 2017 evaluation data was reviewed in full by a single investigator (JAK). Next, the “find” function of Adobe was used to scan the files containing the student evaluations for the following search terms: “map(s),” “concept,” “study,” and “guide” and all related comments were collected into a separate document. The student comments were iteratively grouped to identify the main ideas or codes. A code book was created and used to guide the thematic analysis of the remaining evaluations that were synthesized into major themes and subthemes across the complete data set (440 unique comments).

### Quantitative Survey Design and Analysis

The initial qualitative findings were used to develop a survey for first- and second-year medical students who had completed the Foundational Science module in 2020–2021. Through an iterative process, factors that might influence the adoption of concept mapping related to the FD-maps were hypothesized, and the survey was designed to address each of these factors. Specifically, we postulated that prior exposure to concept mapping, expertise in the content area, ranked importance of FD-maps, and/or map annotation might influence the use of concept mapping in student learning after exposure to the FD-maps (see Appendix for full survey). A total of 31 M2 students (20%) and 39 M1 students (26%) responded to the survey. G*Power software was used to carry out an a posteriori power analysis to determine if there were sufficient responses for analysis [42]. For this study, Fisher’s alpha was set at 0.05 and power set at 0.80. The survey data was subsequently analyzed using IBM SPSS Statistics (Version 28) predictive analytics software. Binary logistic regression was used to model the relationship between predictor variables and binary response variables. To predict the probability of the response variable occurring from a significant predictor, the Wald test was used with Exp(β) representing the odds ratio of the predictor variable effect on the outcome [43]. We also hypothesized that the skeleton nature of the FD-maps would encourage student annotation of maps with linking words, phrases, and the addition of related content. We therefore included questions in the survey addressing the types of annotations that students utilized when using the FD-maps and encouraged students to submit examples of annotated concept maps for analysis.

## Results

### Student Feedback on FD-Maps

**Fig. 1:**
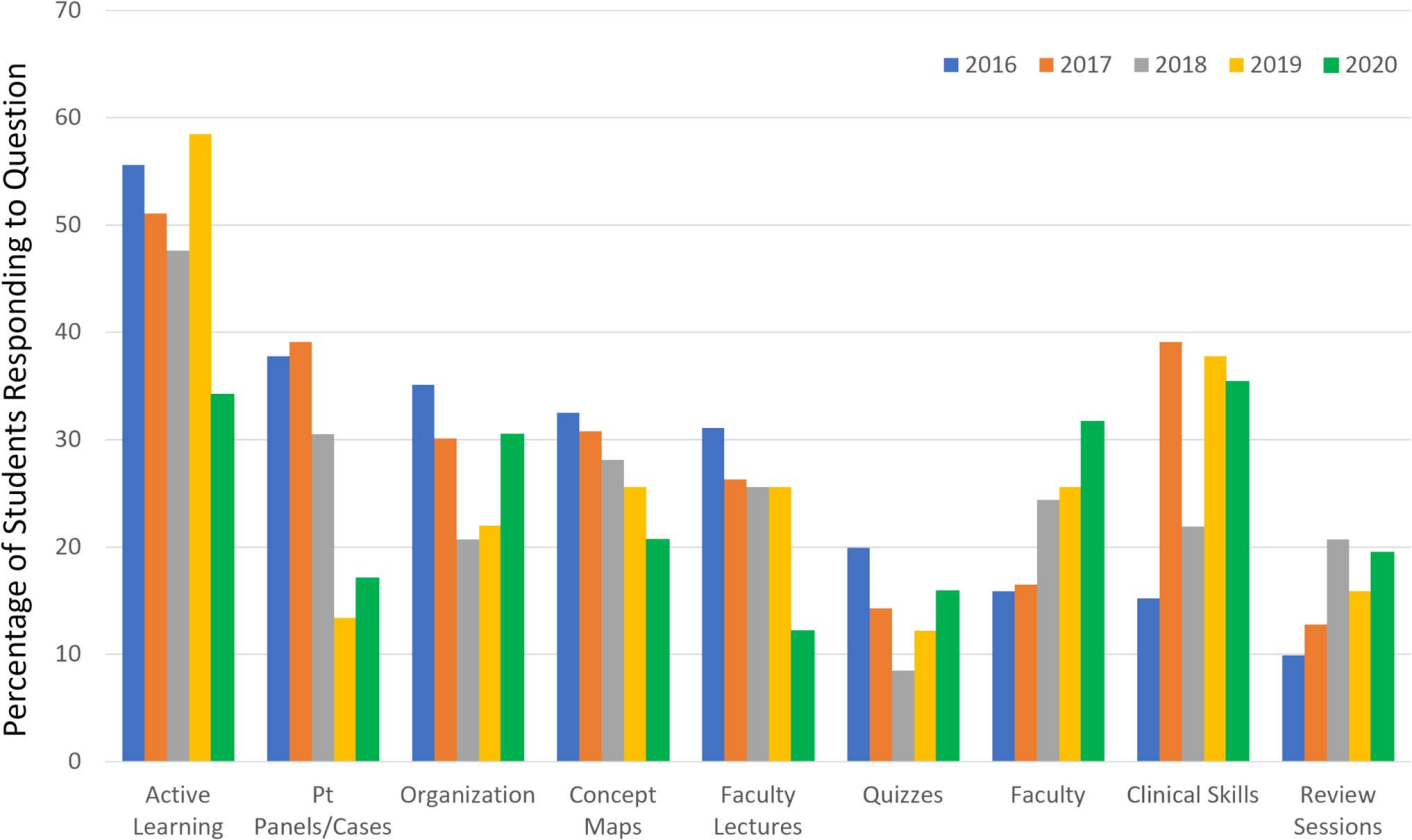
Frequency of student responses to the question “Describe the three best experiences in this module” as a percentage of the total number of student respondents on required end-of-course evaluations from 2016 through 2020

As part of the student evaluations, each cohort completing the Foundational Science module was asked to provide an answer to a variation of the question: “Describe the three best experiences in this module.” Qualitative analysis of the responses shows that FD-maps were consistently noted by the students as within the top 3 to 5 experiences of the module over time, as evidenced by frequency (Fig. 1). The total number of students providing written responses to this question as a part of the evaluation was 151 of 155 students (2016), 133 of 153 students (2017), 82 of 151 students (2018), 82 of 151 students (2019), and 64 of 150 students (2020). The decline in response rate to this question may reflect a higher level of enthusiasm for providing feedback from students experiencing the revised curriculum for the first time in 2016. Other factors that may have contributed to the decline in overall feedback, as well as the relative ranking of the FD-maps over time, include the “novelty” factor, which likely decreased as the maps became an expected component of the curriculum. In addition, the increase in curricular content that occurred in 2018, which included topics with increased complexity such as Immunology, may have contributed to decreased perceived utility of the FD-maps.

Student responses to all free-response questions in student evaluations were subsequently analyzed by thematic analysis using an inductive approach [41], with a total of 440 free-response items included in the analysis. Two major themes related to the use of FD-maps in student learning were identified.

Theme 1—FD-maps facilitated the student perceptions of learning by streamlining note taking and highlighting critical information. Students noted that the FD-maps saved them time, helped guide their learning, and enhanced review of the material. Students wrote that FD-maps “made everything much easier,” “streamline learning,” “facilitated learning of concepts,” allowed them to “spend more time studying instead of making notes,” and made it possible to “keep up with the material better.” Students attributed their improved ability to identify high-yield information to the skeletal FD-maps. Comments such as “highlight important points,” “focus [topics for] studying,” “pick out what’s important,” “Guide learning,” “narrowed what I need to know,” “not get caught up in little details,” and “simplify/clarify what was covered” evidence not only the barriers learners face in retaining core basic science but also the ways FD-maps helped students overcome learning challenges. Finally, FD-maps aided in recall as they provided a review/summary of material. Students commented FD-maps had “helped jogging my memory,” “helped with exams,” “reviewing material for exams,” and were “useful summaries.”

Theme 2—FD-maps brought to light connections between materials that were otherwise less apparent to learners. The students noted that the FD-maps assisted with the integration and organization of the module content. Sample comments include maps “bring everything together” and “connect the dots.” In helping to bring material together, students shared that the FD-maps helped to better “understand relationships” and “visualize material and relationships,” as well as “organize thoughts and notes.”

**Fig. 2:**
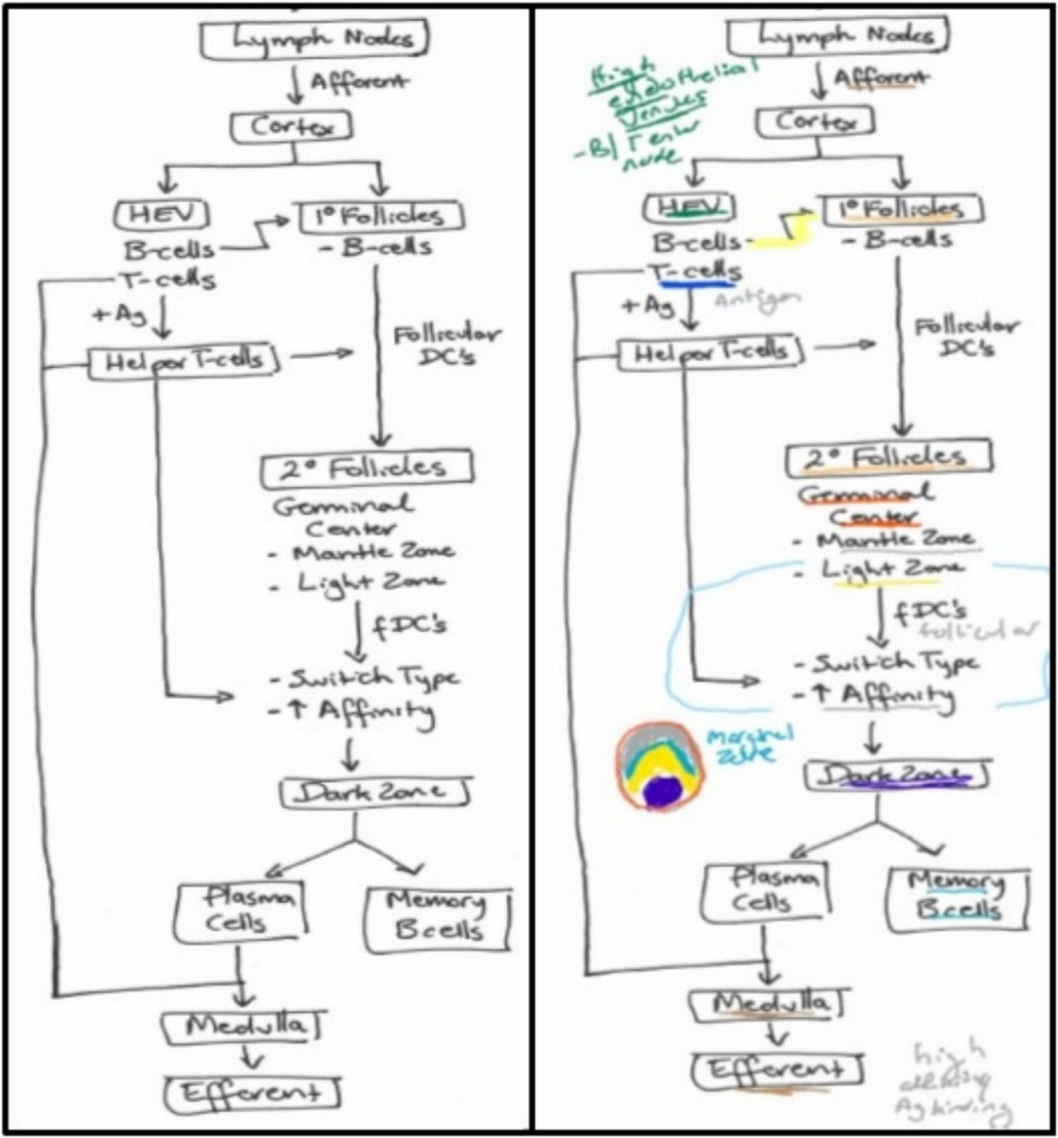
Example of a FD-map (left) and a corresponding annotated map provided by a student (right) illustrating annotations including color-coding of concepts

Additional comments from students suggest that they were taking advantage of the skeleton nature of the maps. For example, students indicated that (they) “can add to in own language” and “annotated/reorganizing them helped me learn.” An example of an annotated map is shown in Fig. 2. There were a few negative comments regarding the FD-maps, but not enough to capture sufficient sentiment. Some students found them hard to follow, which relates to the necessity to individualize concept mapping approaches. Students suggested they would prefer to have been taught how to concept map in advance of using the FD-maps during the module. Additionally, no major differences were noted in the thematic analysis over the course of the 5 years, with two exceptions. First, the response rate was highest in 2016 (153/440 comments), again likely reflecting the introduction of the new curriculum. Second, there was a slight increase in the number of negative comments when the module expanded in 2018 to cover additional topics such as Immunology.

### Survey Data Analysis

The second phase of the sequential mixed methods approach used a project-specific instrument to further assess student use of the FD-maps. Specifically, we were interested in whether prior exposure to concept mapping and/or expertise in the content influenced student use of the FD-maps as a study tool. We intentionally implemented the survey after completion of the Foundational Science module for both the M1 and M2 classes to assess if students adopted concept mapping as a learning strategy after the exposure to the FD-maps. A total of 31 M2 students (20% response rate) and 39 M1 students (26%) responded to the survey. Due to the relatively low response rate, an a posteriori power analysis was conducted to determine whether there were sufficient responses for meaningful analysis. Odds ratios of 2.2 or higher are sufficiently detectable with a sample size of 68; therefore, analysis proceeded.

**Fig. 3:**
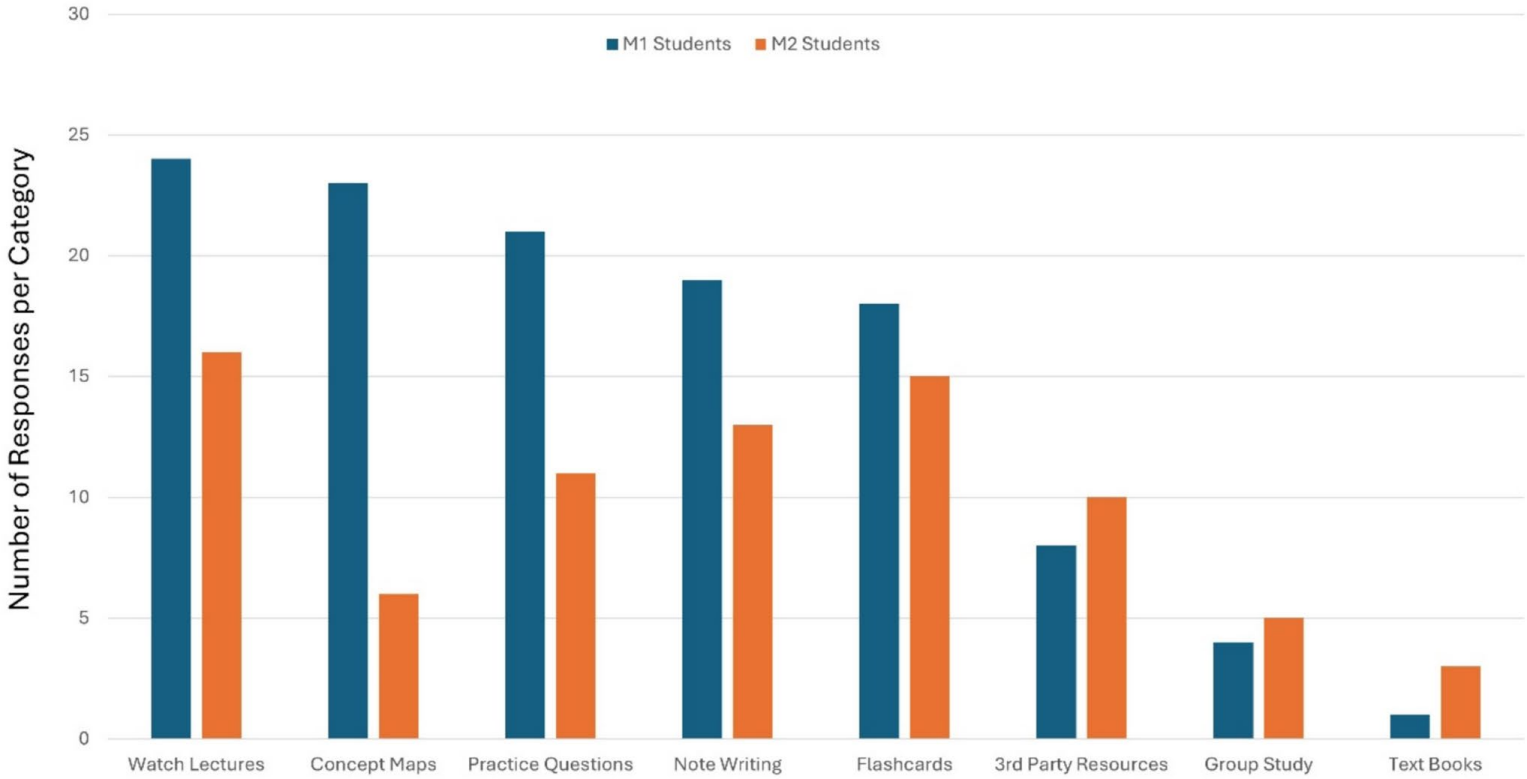
Typical study strategies employed by the survey respondents. Results from M1 (39 students) and M2 (31 students) classes are presented separately for comparison

We first analyzed the types of study strategies employed by the M1 and M2 students. For M1 students, watching lectures, concept maps, practice questions, note writing, and flashcards were popular (Fig. 3), with most students employing more than one method (average of 3.16 strategies per student). In contrast, M2 students employed fewer strategies per student (2.43 per student, *p* = 0.005) with increased use of third-party resources. Regarding concept maps, 27% of all survey respondents had used concept mapping prior to attending medical school. Of those who had not used concept mapping, 45.7% were unfamiliar with the technique while 22.9% of students reported that they did not use concept mapping because it was too time-consuming. An identical percentage (22.9%) of respondents had experienced faculty-developed concept maps in their previous classes. Noticeable in the analysis was that fewer M2 students reported using concept mapping as part of their typical study strategies (Fig. 3) relative to the M1 students. One major difference in the M2 year is the transition to the systems-based curriculum, which may lead some students to use alternate learning strategies. An additional pressure in the M2 year is the concurrent preparation for step 1, which also coincides with the observed increased use of third-party resources in this cohort.

In the context of the Foundational Science module, 67% of students reported the FD-maps to be very important for their study habits using a Likert scale, while only 11.4% indicated that student-derived concept maps were very important for studying for the module (see Table 1). More M1 students rated the FD-maps to be very important when compared to the M2 students, which may reflect their relative proximity to the Foundational Science module. Interestingly, while only 27% of students reported using concept mapping prior to entering the MD program, 74% of students reported using concept mapping as part of their current study strategies after completing the Foundational Science module (Table 1). It is important to note that the extent of student-derived map usage varied, with only 25.6% of M1 and 19.4% of M2 students incorporating concept mapping at either somewhat frequent or very frequent levels. Overall, this does suggest that students were more likely to utilize concept mapping after exposure to the FD-maps, although only 45.7% of survey respondents attributed their current usage of concept mapping to the FD-maps.

**Table 1:** Survey responses to questions related to student use of concept maps

**Survey question**		**Not important at all**	**Minimally important**	**Neutral**	**Somewhat important**	**Very important**
How important is/were the use of faculty-derived concept maps to your study habits?*	**M1**	4	1	0	4	30
	**M2**	2	1	1	10	17
How important were the use of student-derived maps to your study habits?*	**M1**	17	5	9	5	3
	**M2**	9	7	5	5	5
		**Not at all**	**Minimally**	**Equally as frequent as other study methods**	**Somewhat frequently**	**Very frequently**
**How frequently do you currently use concept mapping compared to other studying methods**	**M1**	8	15	6	6	4
	**M2**	10	15	0	3	3

*These two questions specifically referred to the Foundational Science module (edited for brevity)

To gain more insight into the role of FD-maps in student adoption of concept mapping, a binary logistic regression analysis was conducted to investigate if subsequent use of concept maps could be predicted by the self-rated importance of student-derived (SD-maps) or FD-maps in the Foundational Science module. Although the Hosmer-Lemeshow goodness-of-fit suggested the model may not fit (*p* = 0.044) analysis proceeded as the Hosmer-Lemeshow is limited in cases when the estimated probabilities only span a small sub-interval in a small number of covariate patterns [44]. The final predictive model was significant (*χ*^2^(2, 70) = 81.47, *p* < 0.001), explaining approximately 26% of the variance in outcome (Nagelkere *R*^2^ = 0.26) and correctly predicted the outcome 69%, a gain of 15% over the distribution of the dependent variable alone. Importantly, our data showed that student ranking of the importance of FD-maps was a significant predictor of subsequent adoption of mapping (*p* = 0.026). Controlling for student-derived map importance, FD-map importance contributed to the model (*p* = 0.026) and the estimated odds ratio favored a positive relationship more than three times for every one unit increase in the importance of FD-maps. 

As noted in the introduction, the FD-maps were provided as “skeletal” maps, lacking linkage words and providing space for student customization. Our survey showed that 74.3% of students annotated the FD-maps as part of their study strategy, with 14.2% of students adding linking words and phrases, 74.3% adding additional detail, and 48.6% adding content from other lectures and topics. Students also reported adding color-coding, mnemonics, and other pictures to the FD-maps (see Fig. 2 for example). However, only two students submitted sample maps as part of the survey, preventing a more comprehensive analysis of the types of annotations. To determine whether any annotation of the FD-maps predicted future use of self-created concept maps, a binary logistic regression analysis was conducted. The final predictive model (*χ*^2^(1, 70) = 83.41, *p* < 0.001) explained approximately 23% of the variance in outcome (Nagelkere *R*^2^ = 0.23) and correctly predicted the outcome 68%, a gain of 14% compared to the distribution of the dependent variable alone. The predictor annotation was significant (*p* = 0.003) with the estimated odds ratio favoring a positive relationship of adopting concept mapping nearly eleven times when annotation was used. Conducting a similar analysis, we found that student expertise in the subject area was not a factor that contributed to subsequent adoption of concept mapping (*p* = 0.410).

Overall, the mixed methods evaluation of FD-maps indicates positive reactions and outcomes. Once students took ownership of FD-maps, customized with their own gain of knowledge and personalized approach, they were able to creatively transfer concept mapping skills. The final question of our survey was open-ended, asking students to share additional ways that they used the FD-maps for their studying. Students reported adding them to flashcards for use in self-quizzing (4/26 responses), recreating maps from memory to test their knowledge (7/26 responses), and hanging maps together to reveal connections between the maps (2/26 responses). Students noted the utility of the maps for rapid review of content (8/26 responses) and the faculty development of the maps meant that they were trustworthy resources (4/26 responses) with one student noting that the FD-maps were “like reading a textbook without 99% of the weight associated with reading a textbook.”

## Discussion

In this study, we found that FD-maps were highly utilized by the students in the Foundational Science module, with students finding the maps helpful, saving them time and making studying more efficient. The published literature regarding the use of FD-maps in the context of medical education is limited but is consistent in terms of positive responses by students [20, 36, 45, 46]. However, in most studies, the concept maps were restricted to a single session or topic area. This study underscores the utility of FD-maps as the first report that we are aware of to assess FD-maps usage for the entirety of a module. Students noted that an important component of the FD-maps was the ability to focus their studying on critical information. An underlying assumption is that students trusted the FD-maps because they were developed by the faculty as content experts, giving students assurance that the information was accurate. This is consistent with students who generated their own concept maps commenting that they compared these to the FD-maps to ensure that they were capturing high-yield content from the module materials.

A notable finding from the study is that students found that the FD-maps saved time and made studying more efficient, as the time taken to generate concept maps is considered a barrier to their adoption by students [34]. We postulate that it is not apparent to students that learning happens during map generation and that having to construct multiple versions to end up with the final map is a part of the learning process [19, 21, 22, 33, 35]. However, it should be noted that in the preclinical phase of medical education, learners likely prioritize the efficiency of learning over the deeper learning required for effective recall and integration [47]. Despite our intentions to provide a tool enabling students to cognitively manage the vast amount of information associated with the preclerkship phase, the FD-maps may promote more superficial learning [48]. However, our analysis showed that exposure to concept mapping using the FD-maps does influence students to explore concept mapping as a component of their study strategies. Specifically, student ranking of the importance of the FD-maps in their studying did increase their use in subsequent modules and the percentage of students who continued to use concept mapping, even as a minor component of their study strategies, increased after completion of the Foundational Science module.

A second notable finding and key factor that positively related to student adoption of concept mapping was the use of map annotation in the Foundational Science module. Students took advantage of the minimal details on the maps to annotate, adding their own linkage words and visual cues to aid in their studying. Linkage words in concept mapping are indicative of understanding connections and specify the relationship between the concepts [22, 49]. These factors should be considered in promoting student utilization of concept mapping within medical education. However, while FD-maps may partially aid in overcoming barriers for students to adopt concept maps, it should be noted that two-thirds of students who responded to our survey did not use concept mapping to a major extent as part of their routine study strategies. Thus, there is still work to do to encourage students to fully integrate concept mapping into their learning.

One interesting finding from our thematic analysis of the student evaluations was that the FD-maps aided students in organizing concepts and brought to light connections between module content, which is one of the key values typically associated with self-derived maps. This finding may reflect the stage of the learner in the learning process, where explicit illustration of conceptual connections can aid novice learners in the initial acquisition of a mental model. It also supports our premise that FD-maps provided pre-developed schema to assist in the management of the intrinsic load of the module content.

The findings of this study are applicable to medical and health professions educators who may wish to use FD-maps to support their learners, enabling more efficient studying, encouraging cognitive connections, and aiding students in recognizing the value of concept mapping as a study strategy. Nonetheless, the study has limitations. An observational study is not sufficient for causal claims; therefore, results should be interpreted cautiously. Specifically, as FD-maps were included for all module learning materials, and as part of a major curricular reform from a discipline to systems-based curriculum, and the module itself revised to incorporate cognitive learning theory, it would be difficult to ascribe any learning outcomes solely to the FD-maps. In addition, part of the data collection timeframe overlaps with the COVID-19 pandemic and the shift to remote emergency online module delivery modes, which may have influenced respondents. Finally, although responses were deemed sufficient through power calculation, low response rates in the quantitative phase may be a cause for a replicated investigation.

## Conclusion

Our study supports the utility of faculty-derived concept maps to aid in student perception of learning. In addition, our findings suggest that a curriculum structure that supports the use of FD-maps can enhance student adoption of concept mapping as a study strategy. Allowing space for student annotation and customization within the FD-maps may further enhance the adoption of concept mapping.

## Data Availability

The data that support the findings of this study are available from the corresponding author, JAK, upon reasonable request.

## Abbreviation

FD-maps: Faculty-derived maps
